# Mental illness-related stigma and its associated factors among primary health care professionals in rural China

**DOI:** 10.3389/fpsyt.2025.1519527

**Published:** 2025-05-30

**Authors:** Yi-Yue Yang, Cong Wang, Jia Cai, Yun-Fei Mu, Lie Zhou, Yi-Zhou Wang, Tian-Ming Zhang, Xin-Yi Zhao, Ming Li, Wei Luo, Jian-Jun Luo, Yin-Ling IreneWong, Lawrence H. Yang, Siu-Man Ng, Mao-Sheng Ran

**Affiliations:** ^1^ Mental Health Center, West China Hospital, Sichuan University, Chengdu, Sichuan, China; ^2^ Institute of Psychiatry, West China Hospital, Sichuan University, Chengdu, Sichuan, China; ^3^ Department of Counselling and Psychology, Hong Kong Shue Yan University, Hong Kong, China; ^4^ Department of Social Work, Shanghai University, Shanghai, China; ^5^ School of Health Humanities, Peking University, Beijing, China; ^6^ Xinjin Second People’s Hospital, Chengdu, China; ^7^ Chongqing Mental Health Center, Chongqing, China; ^8^ School of Social Policy & Practice, University of Pennsylvania, Philadelphia, PA, United States; ^9^ Department of Social and Behavioral Sciences, New York University, New York, NY, United States; ^10^ Department of Social Work and Social Administration, The University of Hong Kong, Hong Kong, China

**Keywords:** primary health care professionals, mental illness, stigma, influencing factors, rural China

## Abstract

**Background:**

Primary health care professionals (PHCPs) play a key role in the workforce of community mental health services in rural China. This study aimed to explore the mental illness-related stigma and its associated factors among PHCPs in rural communities.

**Methods:**

This study collected the data from 247 PHCPs in 10 township health service centers in Xinjin District, Chengdu, Sichuan Province, China from November to December 2023. The Mental Illness: Clinicians’ Attitudes (MICA) was used to assess the mental illness-related stigma. Demographic and stigma-related psychological scales were compared between PHCPs with and without mental illness-related stigma. Correlation and binary logistic regression analyses were performed.

**Results:**

There were 155 PHCPs (62.8%) with mental illness-related stigma, and the mean score of MICA was 50.68 ± 8.08. PHCPs with mental illness-related stigma had significantly lower mean scores of the Mental Health Knowledge Schedule (MAKS), the Reported and Intended Behavior Scale (RIBS), the 5-item Contact Scale (CQTS), and the 5-item Contact Quality Scale (CQLS) (p=0.001, p<0.001, P=0.041, P<0.001), and higher mean scores of the Social Distance Scale (SDS) (p<0.001) than those without mental illness-related stigma. Binary logistic regression analysis showed that PHCPs’ work experience (β=0.080, 95%CI=1.002~1.170, p=0.044) and scores of SDS (β=0.169, 95%CI=1.056~1.328, p=0.004) had significantly positive impact on the mental illness-related stigma, and the scores of MAKS (β=-0.082, 95%CI=0.850~0.998, p=0.045) and RIBS (β=-0.131, 95%CI=0.783~0.983, p=0.024) had significantly negative impact on the mental illness-related stigma.

**Conclusions:**

The PHCPs have severe mental illness-related stigma in rural China, and the associated factors include work experience, mental health knowledge, behavioral discrimination, and social distance towards people with mental illness. The results of this study are crucial for development of anti-stigma intervention among PHCPs in rural communities.

## 1 Introduction

Stigma, which refers to the shame that individuals feel about having a disease, as well as negative social attitudes towards illness (e.g., stereotypes, prejudices, and attitudes) and behaviors towards the disease (1), is a complex and pervasive phenomenon in the field of mental health (2, 3). Evidence shows that mental illness-related stigma and discrimination may cause more negative impacts among persons with mental illness than the mental illness itself (4). It not only affects patients’ willingness to seek help and treatment but also aggravates social discrimination against people with mental illness (5, 6).

In rural China, because of the limited mental health professional services, primary health care professionals (PHCPs) (including medical staff, nursing staff, pharmaceutical staff, technicians, etc.) are an important labor force in community mental health services (7). Although the government has increased its investment in the field of mental health in recent years, these investments face multiple challenges in practice, including economic, cultural and medical resources, PHCPs in rural areas may have severe mental illness-related stigma (8). In many rural communities, there may be a lack of understanding and awareness about mental health issues, leading to misconceptions (including dangerousness and unpredictability) and discrimination against those with mental illnesses. PHCPs may not have adequate training or resources to effectively address the needs of individuals with severe mental illnesses (9). This will affect the service attitude and service quality of PHCPs to persons with mental illness and their families, which is not conducive to the treatment and rehabilitation of patients (10, 11). Moreover, the notion of collectivism in China also lead to persons with mental illness more easily internalize the public stigma against themselves (12).

The mental illness-related stigma among PHCPs demonstrates significant correlations with sociodemographic and psychological determinants. From a sociodemographic perspective, the relationship between age and mental illness-related stigma reveals context-dependent patterns. Studies indicate that older clinicians tend to exhibit heightened stigmatization toward psychiatric disorders, particularly toward individuals with schizophrenia, potentially attributable to generational biases or reduced patient exposure (13). However, frequent clinical interactions with patients may mitigate stigma among senior practitioners, suggesting a complex interplay between chronological age and experiential factors (14). While extended professional tenure itself does not necessarily correlate with elevated stigma, inadequate continuing education or suboptimal patient interactions may exacerbate negative attitudes (15). Notably, some research identifies that stigmatizing attitudes remain independent of demographic variables like gender and age, being predominantly influenced by specialized training and clinical experience (16). The most consistent determinant across studies is educational attainment: lower educational levels show strong associations with intensified stigma. For instance, paraprofessionals (e.g., assistants or technicians with limited formal education) demonstrate significantly greater stigmatizing tendencies compared to psychologists or physicians (14). Particularly, individuals with only primary education exhibit markedly higher stigma scores than those with advanced degrees (17). Furthermore, studies have identified elevated depression-related stigma among male PHCPs, which may be associated with gender-specific stereotypes regarding mental health issues or societal role expectations (18). Conversely, population-based research demonstrates that females in the general population exhibit higher levels of stigmatization toward mental disorders (19). Regarding psychological aspects, prior research shows that the associated factors of mental illness-related stigma of medical staff include mental health knowledge, behavioral discrimination, social distance, positive contact (20–22). Medical personnel may exhibit a lack of understanding, mistrust, or even discrimination towards patients, primarily stemming from inadequacies in their relevant professional knowledge and clinical experience. The attitudes and behaviors of this lack of understanding, mistrust, and even discrimination not only aggravate the stigma of patients but also may affect the doctor-patient relationship and the treatment effect of patients (23). Some studies have found that although psychiatric staff have a more positive attitude towards patients with mental illness, they always want to keep a greater distance from patients (especially patients with schizophrenia) (24, 25).

The existing literature exhibits three critical gaps: First, most studies focus on the stigma experienced by patients or family members, while neglecting the impact of healthcare providers’ own stigmatizing attitudes on service quality (26, 27); Second, studies targeting medical professionals have mainly been conducted in urban medical institutions or qualitative studies in rural areas, lacking direct quantitative evidence indicating that phcp in rural China is a unique population (28–30). Third, although factors like mental health knowledge, behavioral discrimination, social distance, contact, and social factors are known to correlate with stigmatization, it remains unclear whether rural-specific structural factors (e.g., scarcity of training resources, entrenched cultural beliefs) modify these relationships (31, 32). Therefore, this study aims to: (1) quantitatively assess the prevalence and severity of mental illness-related stigma among PHCPs in rural China, and (2) examine the associations between PHCPs’ sociodemographic and psychological factors and mental illness-related stigma levels. We propose the following hypotheses: (1) Mental illness-related stigma among rural Chinese PHCPs demonstrates a high prevalence and significant severity; (2) Among sociodemographic factors, the older, longer work experience and lower education level are positively associated with higher mental illness-related stigma levels while gender may be related to the level of mental illness related stigma; (3) Regarding psychological factors, poorer mental health knowledge, stronger behavioral discrimination tendencies toward patients, greater desired social distance, and less contact quantity and quality with individuals with mental illness are significantly linked to elevated mental illness-related stigma levels. The research results will directly serve three goals: 1) Supplement the research on stigma in the field of mental health services in rural primary medical institutions; 2) Provide a scientific basis for designing targeted anti-stigmatization intervention programs; 3) Emphasize the importance of enhancing service capabilities by reducing the mental illness-related stigma of PHCPs, optimizing the rural mental health service system, and ultimately improving the accessibility of treatment and the quality of rehabilitation for patients with mental disorders.

## 2 Materials and methods

### 2.1 Participants

From November to December 2023, a consecutive sample of 256 PHCPs (121 doctors, 75 nurses, 6 Psychiatric professionals, 45 others) were recruited through onsite invitations at weekly staff meetings from 10 township health service centers in Xinjin County, Chengdu City, Sichuan Province, China. Inclusion criteria for participants: (1) PHCPs working full-time (>30hrs/week as defined by local health administration standards) in the township health service centers (e.g., hospital or clinic), (2) age range 18–65 years old, with birth dates cross-checked against government-issued ID cards, (3) self-reported never diagnosed with from mental illness, confirmed through monthly health checkup records, and (4) Mandarin Chinese proficiency to understand and cooperate with the investigation. Exclusion criteria include: (1) incomplete questionnaire submission (>20% missing data) as determined by real-time quality checks during data collection and (2) current psychiatric diagnosis documented in medical records, and (3) temporary staff or rotating trainees with <1 months service tenure. Potential participants were approached by trained research coordinators during pre-scheduled 30-minute recruitment sessions, where study objectives and procedures were explained using standardized slides. The recruitment rate was 96.5% (247/256 eligible PHCPs), with refusals primarily due to scheduling conflicts (n=8) or personal reluctance (n=1). Participants received 30 yuanupon completion as compensation for time spent.

Demographic data and clinical psychological questionnaires were completed via paper-and-pencil format during scheduled work breaks by all participants under the supervision of trained research assistants (graduate students certified in ethical data collection), achieving 100% response rate with no missing data. Each questionnaire underwent dual verification by independent raters using a standardized checklist. All participants gave written informed consent after receiving standardized oral/written study information. The study was approved by the Human Research Ethics Committee (HREC) of the University of Hong Kong (No: EA2001046).

### 2.2 Measures

#### 2.2.1 Demographic data

We used a self-developed demographic questionnaire to collect demographic data, including gender, age, education level, occupation, work experience, marital status, family size, annual income, whether to have contact with people with mental illness.

#### 2.2.2 Mental illness-related stigma

The Mental Illness: Clinicians’ Attitudes (MICA) was used to assess the mental illness-related stigma among PHCPs. The MICA has been used to assess the attitudes of clinicians and other medical professionals towards persons with mental illness (33). It consists of 16 items, each consisting of six options on a 6-point scale, 1 = strongly agree, 2 = agree, 3 = somewhat agree, 4 = somewhat disagree, 5 = disagree, and 6 = strongly disagree. The scores of each item are summed to obtain the total score, ranging from 16 to 96. We used a median of 48 as the cutoff point, defined a score of 16 to 48 as having no mental illness-related stigma and 49 to 96 as having mental illness-related stigma (34). The higher the score, the greater the mental illness-related stigma. MICA has been proven to have good reliability and validity among medical students and healthcare workers (35). The Cronbach’s Alpha of the Chinese version of MICA used in this study was 0.687.

#### 2.2.3 Mental health knowledge

The Mental Health Knowledge Schedule (MAKS) was used to assess participants’ stigma-related mental health knowledge (36). The MAKS contains 12 items, and 6 items are related to the stigma-related mental health knowledge, including seeking help, recognition and support, employment, treatment, and rehabilitation, and another 6 are opinions of what disease belongs to mental illness. Each item is on a 5-point Likert scale, 1 = strongly disagree, 2= somewhat disagree, 3= neither agree nor disagree, 4= somewhat agree, and 5 = strongly agree. The higher the score, the more knowledge of mental health. The scale has been proven to have good reliability and validity among community health staff in China (37). The Cronbach’s Alpha of the Chinese version of MAKS used in this study was 0.700.

#### 2.2.4 Behavior discrimination

The Reported and Intended Behavior Scale (RIBS) was used to assess participants’ behavioral discrimination related to persons with mental illness (38). It consists of eight items, of which items 1–4 assess the incidence of behaviors related to persons with mental illness, including the three options “Yes”, “No” and “Don’t know”. Items 5–8 assess the willingness to engage in behaviors related to persons with mental illness. It is 5-point Likert scale, 1 = strongly disagree, 2= somewhat disagree, 3= neither agree nor disagree, 4= somewhat agree, and 5 = strongly agree. Scores from 5 to 8 items are summed to give a total score, ranging from 4 to 20, with higher scores indicating lower level of behavioral discrimination against persons with mental illness. The Chinese version of RIBS has strong consistency reliability (38). The Cronbach’s Alpha of the Chinese version of RIBS used in this study was 0.765.

#### 2.2.5 Social distance

The Social Distance Scale (SDS) was used to assess participants’ social distance from persons with mental illness (39). SDS contains 7 items, each item has four options: 0 = completely willing, 1 = willing, 2 = won’t, 3 = completely not. The total scores are from 0 to 21 points, the higher the score indicates the more inclined to keep a larger social distance with persons with mental illness. The Cronbach’s Alpha of the Chinese version of SDS used in this study was 0.870.

#### 2.2.6 Contact quantity

The 5-item Contact Scale (CQTS) was used to assess assessed the amount of contact participants had with people with mental illness at work (CQTS-Work), with people with mental illness in their neighborhood (CQTS-Neighbors), with friends and relatives of people with mental illness (CQTS-Relatives and Friends), with non-positive contact with people with mental illness (CQTS-Non-positive contact), and with people with mental illness at home (CQTS-Home) (40). Each item is a 7-point Likert scale (1= no contact at all, 7= frequent contact). The scores of all items were summed to obtain the total score, with higher total scores indicating more frequent contact with persons with mental illness. The Cronbach’s Alpha of the Chinese version of CQTS used in this study was 0.890.

#### 2.2.7 Contact quality

A 5-item Contact Quality Scale (CQLS) was used to assess the contact quality between participants and persons with mental illness in five aspects: whether the contact was equal, voluntary, intimate, pleasant and cooperative (40). Each item was rated on a seven-point Likert scale, 1= strongly disagree, 2= disagree, 3= somewhat disagree, 4= neither agree nor disagree, 5= somewhat agree, 6= agree, and 7= strongly agree. All items combined to get the total score, the higher the score, the higher the quality of contact with persons with mental illness. The Cronbach’s Alpha of the Chinese version of CQLS used in this study was 0.870.

#### 2.2.8 Depressive symptoms

The 9-item version of the Patient Health Questionnaire (PHQ-9) was used to assess participants’ depressive symptoms. The scale has been commonly used and is considered the most reliable tool to screen for depressive symptoms (41, 42). Each item is assessed on how often you have been bothered by the symptoms in the past two weeks. There are four options, 0= not at all, 1= a few days, 2= more than half the days, and 3= almost every day. The 9 items were added together to give an overall score, with higher scores indicating more severe depressive symptoms. The Cronbach’s Alpha of the Chinese version of PHQ-9 used in this study was 0.875.

#### 2.2.9 Anxiety symptoms

The Generalized Anxiety Disorder Self-rating Scale (GAD-7) was used to assess the anxiety symptoms of the participants, which is a widely used scale to measure anxiety symptoms (43). It consists of seven items, each item is assessed on how often they have the symptoms in the past two weeks. There are four options, 0= not at all, 1= a few days, 2= more than half the days, and 3= almost every day. The total score was obtained by direct addition, with higher scores indicating more severe anxiety symptoms. The Cronbach’s Alpha of the Chinese version of GAD-7 used in this study was 0.901.

### 2.3 Statistical analysis

The SPSS software (version 26) was used for statistical analysis of the data of PHCPs. 1). Data cleaning and pretreatment, the missing value, and the mean value interpolation method were adopted to fill. 2). T-test and one-way ANOVA were used to test whether the mental illness-related stigma differed by demographic characteristics of the participants, and *post hoc* multiple tests were performed if the results of one-way ANOVA were significantly different. Participants were divided into two groups (cut-off point=48), G1= without mental illness-related stigma, and G2= with mental illness-related stigma. T-test and Chi-square tests were used to explore the differences in socio-demographic and psychological scales between the two groups. 3). Pearson correlation analysis was used to analyze the correlation between mental illness-related stigma and various related factors. 4). Binary logistic regression analysis was conducted with mental illness-related stigma as the dependent variable and other related factors as independent variables to explore the associated factors of mental illness-related stigma. The positive results were included in the ROC curve to explore the suggestive efficacy of the associated factors. P < 0.05 was considered a statistically significant difference.

## 3 Results

### 3.1 Demographic characteristics and mental illness-related stigma of participants

This study included 247 PHCPs (**Table 1**), most of them were females (74.5%), aged 18 to 39 years old (55.1%), with junior college degree (53.8%), and doctors (49.0%). About half of the participants had more than 15 years of work experience (52.2%), got married (83.4%), had a family size > 4 (58.3%), and annual income > 100000 RMB (59.5%). In addition, majority of them did not contact with persons with mental illness in the past month (67.2%).

**Table 1:** Demographic characteristics and mental illness-related stigma of PHCPs.

Characteristic	N (%)	MICA (N=247)				Group		
		Mean± SD	Test statistic	*Post-hoc*	p	G1 (N=92)	G2 (N=155)	p
Gender			T=0.756		0.450			0.872
Male	63 (25.5%)	51.35**±**8.178				24 (26.1%)	39 (25.2%)	
Female	184 (74.5%)	50.46**±**8.061				68 (73.9%)	116 (74.8%)	
Age			T=3.718		<0.001			0.013
18–39	136 (55.1%)	49.00**±**7.954				60 (65.2%)	76 (49.0%)	
40-65	111 (44.9%)	52.75**±**7.790				32 (34.8%)	79 (51.0%)	
Educational level			F=3.436		0.034			0.340
≤High School/Technical secondary school (1)	39 (15.8%)	52.90**±**8.509		1>3		14 (15.2%)	25 (16.1%)	
Junior college (2)	133 (53.8%)	51.03**±**7.868				45 (48.9%)	88 (56.8%)	
Undergraduate (3)	75 (30.4%)	48.92**±**7.979				33 (35.9%)	42 (27.1%)	
Occupations			F=2.613		0.052			0.078
Doctors	121 (49.0%)	51.11**±**7.901				46 (50.1%)	75 (48.5%)	
Nurses	75 (30.4%)	49.69**±**8.022				28 (30.4%)	47 (30.3%)	
Psychiatrists	6 (2.4%)	43.50**±**5.541				5 (5.4%)	1 (0.6%)	
Others	45 (18.2)	52.16**±** 8.482				13 (14.1%)	32 (20.6%)	
Work Experience (years)			T=3.526		0.001			0.002
< 15	118 (47.8%)	48.83**±**8.196				56 (60.9%)	62 (40.0%)	
≥15	129 (52.2%)	52.38**±**7.624				36 (39.1%)	93 (60.0%)	
Marital status			F=4.212		0.016			0.054
Unmarried (1)	29 (11.7%)	46.76**±**9.800		1<2		15 (16.3%)	14 (9.0%)	
Married (2)	206 (83.4%)	51.30**±**7.729				70 (76.1%)	136 (87.8%)	
Divorced (3)	12 (4.9%)	49.67**±**7.402				7 (7.6%)	5 (3.2%)	
Family size			T=1.319		0.189			0.482
< 4	103 (41.7%)	49.88**±**8.497				41 (44.6%)	62 (40.0%)	
≥4	144 (58.3)	51.26**±**7.754				51 (55.4%)	93 (60.0%)	
Annual income (RMB)			T=-0.169		0.866			0.947
< 100000	100 (40.5%)	50.79**±**7.856				37 (40.2%)	63 (40.6%)	
≥100000	147 (59.5%)	50.61**±**8.261				55 (59.8%)	92 (59.4%)	
Contact history of people with mental illness (within one month)			T=-1.775		0.077			0.028
Yes	81 (32.8%)	49.38**±**8.937				38 (41.3%)	43 (27.7%)	
No	166 (67.2%)	51.32**±**7.581				54 (58.7%)	112 (72.3%)	

G1, without mental illness-related stigma; G2, with mental illness-related stigma.

The mental illness-related stigma was significantly different among different age, education level, work experience, and marital status groups (p<0.001, T=3.7186; p=0.034, F=3.436; p=0.001, T=3.526; p=0.016, F=4.212). The 40–65 years group had significantly higher mean scores of stigma than the 18–39 years group (p<0.001, T=3.719), and the work experience ≥15 years group had significantly higher mean scores of stigma than the work experience < 15 years group (p=0.001, T=3.526). Participants with high school/technical secondary degree had significantly higher mean scores of mental illness-related stigma than those with undergraduate degree (p=0.034, F=3.436), and the married group had significantly higher mean scores of stigma than the unmarried group (p=0.016, F=4.212). In the chi-square test, age, length of work experience, and contact history with mental illness (within 1 month) were significantly different between G1 group and G2 group (p=0.013, p=0.002, p=0.028).

### 3.2 Psychological scales of participants

**Table 2** shows the psychological scales of PHCPs. In the t-test and chi-square test, participants without mental illness-related stigma had significantly higher mean scores or level of MAKS, RIBS, CQTS-Work, and CQLS than those with mental illness-related stigma (p=0.001, p<0.001, P=0.041, P<0.001), and participants without mental illness-related stigma had significantly lower mean score or level of MICA and SDS than those with mental illness-related stigma (p<0.001, P<0.001). There was no significant difference of mental illness-related stigma between the two groups in other variables (all p>0.05).

**Table 2:** Stigma related psychological scales of PHCPs.

Characteristic			N (%)	Range	Mean	SD	Group		p
							G1 (N=92)	G2 (N=155)	
MAKS				33-56	46.11	4.104	47.24**±**3.375	45.44**±**4.355	0.001
MICA				27-70	50.68	8.084	42.24**±**4.708	55.70**±**4.837	<0.001
RIBS				4-20	11.65	3.375	13.13**±**3.430	10.77**±**3.025	<0.001
	1.Are you currently living with, or have you ever lived with, someone with a mental health problem?								0.587
		Yes	12 (4.86%)				3 (3.3%)	9 (5.8%)	
		No	226 (91.50%)				86 (93.5%)	140 (90.3%)	
		Don’t know	9 (3.64%)				3 (3.3%)	3 (6%)	
	2.Are you currently working with, or have you ever worked with, someone with a mental health problem?								0.583
		Yes	16 (6.48%)				5 (5.4%)	11 (7.1%)	
		No	210 (85.02%)				81 (88.0%)	129 (83.2%)	
		Don’t know	21 (8.50%)				6 (6.5%)	15 (9.7%)	
	3.Do you currently have, or have you ever had, a neighbour with a mental health problem?								0.540
		Yes	68 (27.53%)				25 (27.2%)	43 (27.7%)	
		No	163 (65.99%)				63 (68.5%)	100 (64.5%)	
		Don’t know	16 (6.48%)				4 (4.3%)	12 (7.7%)	
	4. Do you currently have, or have you ever had, a close friend with a mental health problem?								0.330
		Yes	37 (14.98%)				13 (14.1%)	24 (15.5%)	
		No	192 (77.73%)				75 (81.5%)	117 (75.5%)	
		Don’t know	18 (7.29%)				4 (4.3%)	14 (9.0%)	
SDS				0-21	12.71	3.480	11.11**±**3.320	13.66**±**3.224	<0.001
CQTS				5-35	11.04	7.850	11.88**±**8.302	10.55**±**7.553	0.198
	Work			1-7	2.74	2.186	3.11**±**2.323	2.52**±**2.078	0.041
	Neighbors			1-7	1.93	1.675	2.00**±**1.703	1.89**±**1.662	0.620
	Relatives and Friends			1-7	1.93	1.689	2.00**±**1.816	1.89**±**1.614	0.623
	Non-positive contact			1-7	1.96	1.698	2.05**±**1.842	1.90**±**1.611	0.500
	Home			1-7	2.48	2.166	2.72**±**2.350	2.34**±**2.043	0.188
CQLS				5-35	24.47	6.107	26.61**±**5.778	23.20**±**5.957	<0.001
PHQ-9				0-25	3.05	3.724	2.75**±**4.205	3.23**±**3.408	0.333
GAD-7				0-19	2.17	3.011	1.80**±**3.068	2.38**±**2.966	0.146

G1, without mental illness-related stigma; G2, with mental illness-related stigma.

### 3.3 Correlation between participants’ mental illness-related stigma and other variables

The results of the correlation analysis are summarized in **Table 3**. The scores of MICA were significantly positively correlated with participants’ age, work experience, and the scores of SDS (p<0.01), and significantly negatively correlated with the scores of MAKS, RIBS, CQTS-Work, and CQLS (p<0.05 or p<0.01).

**Table 3:** Correlation between participants’ mental illness-related stigma and other variables.

Variable	1	2	3	4	5	6	7	8
1. MICA	1							
2. Age	0.292^**^	1						
3. Work Experience	0.287^**^	0.896^**^	1					
4. MAKS	-0.210^**^	-0.045	-0.002	1				
5. RIBS	-0.400^**^	-0.084	-0.090	0.218^**^	1			
6. SDS	0.433^**^	0.091	0.096	-0.168^**^	-0.557^**^	1		
7. CQTS-Work	-0.138^*^	0.091	0.133^*^	0.296^**^	0.184^**^	-0.208^**^	1	
8. CQLS	-0.351^**^	-0.081	-0.050	0.246^**^	0.455^**^	-0.446^**^	0.381^**^	1

MICA, The Mental Illness: Clinicians’ Attitudes; MAKS, The Mental Health Knowledge Schedule; RIBS, The Reported and Intended Behavior Scale; SDS, The Social Distance Scale; CQTS, The 5-item Contact Scale; CQLS, A 5-item Contact Quality Scale.

### 3.4 Associated factors of the mental illness-related stigma among participants

Binary logistic regression analysis showed that the model fitted well (**Table 4**), with R²=0.286 > 0.2, indicating that the results of this calculation were reliable. The results showed that participants’ work experience (β=0.080, 95%CI=1.002~1.170, p=0.044) and scores of SDS (β=0.169, 95%CI=1.056~1.328, p=0.004) had significantly positively impact on the mental illness-related stigma. The scores of MAKS (β=-0.082, 95%CI=0.850~0.998, p=0.045) and RIBS (β=-0.131, 95%CI=0.783~0.983, p=0.024) had significantly negatively impact on the mental illness-related stigma.

**Table 4:** Associated factors of mental illness-related stigma of participants.

Variable	B	SE	Wald X^2^	p	OR	95%CI		R²
						Lower	Upper	
Age	-0.021	0.036	0.331	0.565	0.979	0.912	1.052	0.286
Work Experience	0.080	0.040	4.040	0.044	1.083	1.002	1.170	
MAKS	-0.082	0.041	4.036	0.045	0.921	0.850	0.998	
RIBS	-0.131	0.058	5.097	0.024	0.877	0.783	0.983	
SDS	0.169	0.058	8.327	0.004	1.184	1.056	1.328	
CQLS	-0.057	0.072	0.623	0.430	0.945	0.820	1.088	

MAKS, The Mental Health Knowledge Schedule; RIBS, The Reported and Intended Behavior Scale; SDS, The Social Distance Scale; CQTS, The 5-item Contact Scale; CQLS, A 5-item Contact Quality Scale.

**Figure 1:**
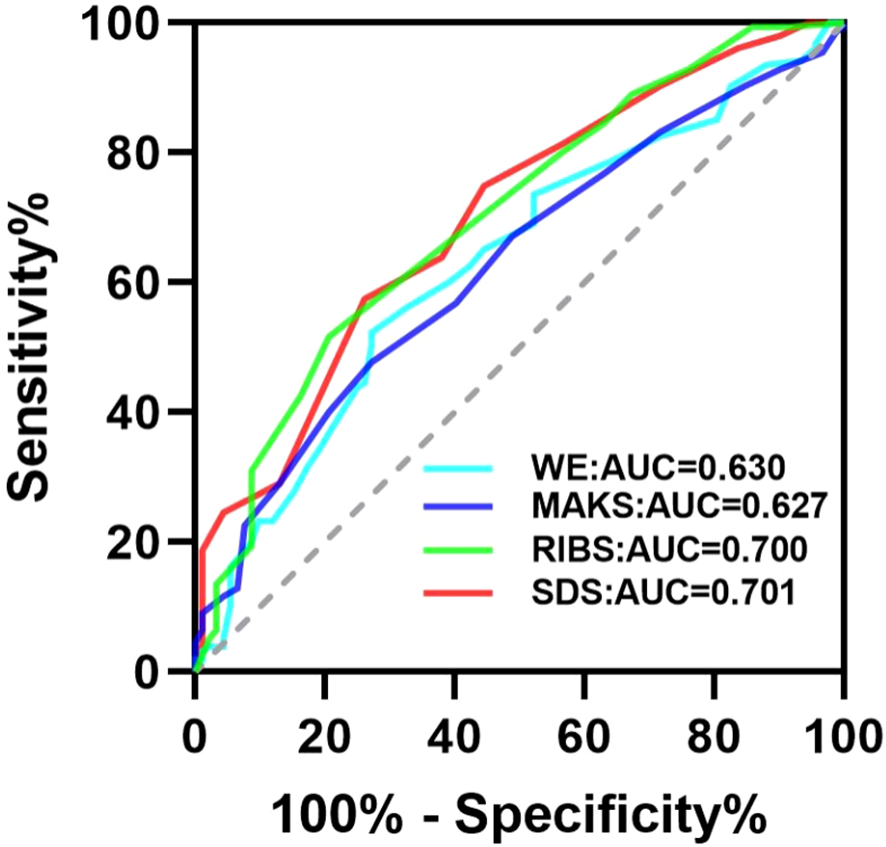
Prompt power of candidate variables for the presence or absence of mental illness-related stigma. WE, Work Experience.

The ROC curve is shown in **Figure 1**. Participants’ work experience (AUC=0.630) and the scores of MAKS (AUC=0.627) had prompt value for mental illness-related stigma, but the prompt effect was low. Participants’ scores of RIBS (AUC=0.700) and SDS (AUC=0.701) had high prompt effect for mental illness-related stigma. Truncation values of work experience, MAKS, RIBS, and SDS were 16.5, 45.5, 11.5, and 13.5, respectively (**Table 5**).

**Table 5:** The value of the ROC curve.

Variable	AUC	95%CI		Youden index	Truncation value	sensitivity	specificity
		Lower	Upper				
Work Experience	0.630	0.559	0.702	0.251	16.500	0.523	0.728
MAKS	0.627	0.557	0.697	0.206	45.500	0.523	0.272
RIBS	0.700	0.633	0.767	0.310	11.500	0.484	0.207
SDS	0.701	0.634	0.767	0.313	13.500	0.574	0.739

MAKS, The Mental Health Knowledge Schedule; RIBS, The Reported and Intended Behavior Scale; SDS, The Social Distance Scale.

## 4 Discussion

To the best of our knowledge, this is the first quantitative study to investigate mental illness-related stigma and its associated factors among PHCPs in rural China. This study measured the demographic characteristics and stigma-related psychological scales of 247 PHCPs to explore the mental illness-related stigma experienced by PHCPs in rural China. It is essential to reduce PHCPs’ mental illness-related stigma, and improve the quality of community mental health services in rural areas.

The results of this study showed that most PHCPs in rural China had mental illness-related stigma (62.8%), consistent with previous qualitative research on mental illness-related stigma among PHCPs in rural China (30), a rate significantly higher than that observed among healthcare providers (HCPs) in developed countries (40%-55%) (44) — a disparity that may be closely linked to insufficient medical resources, low mental health knowledge rates, and entrenched cultural perceptions in rural areas (27). These findings align with the characteristic pattern of heightened biases and misconceptions regarding mental disorders among PHCPs (45) and are consistent with previous studies (8, 9, 30). The mental illness-related stigma is manifested not only in the underestimation of patients’ capabilities, but also permeates clinical practice through reduced willingness to provide services, increased misdiagnosis rates, and treatment delays (46). This discriminatory attitude may exacerbate patient stigma through “labeling” and “differential treatment” (47), thereby creating a vicious cycle of “stigma-treatment avoidance-functional deterioration” that hinders help-seeking behaviors and rehabilitation processes (48).

In terms of social demographic characteristics, this study showed that PHCPs with and without mental illness-related stigma had significantly differences of age, work experience, and contact history of persons with mental illness (within one month) while the gender difference was not statistically significant. Specifically, the older, with longer work experience, ≤ high School/Technical secondary school degree, and married PHCPs showed a more severe tendency towards mental illness-related stigma, and both age and work experience were significantly positively correlated with their mental illness-related stigma. The binary logistic regression analysis indicated that work experience had a significant positive impact on mental illness-related stigma among PHCPs, but ROC curve analysis demonstrated its limited predictive efficacy. When the work experience cut-off value was set at 16.5 years, PHCPs exceeding this threshold exhibited a higher risk of stigmatization, suggesting that long-term grassroots service experience may serve as a biosocial marker for mental illness-related stigma accumulation. Regarding age and work experience, this result is consistent with many previous studies (13, 49). The possible reasons may include that as the growth of the age, the social information and ideas that individuals are exposed to will gradually solidify (50), the traditional bias and misunderstanding on mental illness may be more entrenched. While frequent clinical interactions with patients may reduce mental illness-related stigma among experienced clinicians (14, 51), negative work experiences may also accumulate over time, such as the treatment difficulties of persons with mental illness and social discrimination, which may aggravate their mental illness-related stigma. Similarly, as their work experience increases, PHCPs may experience burnout, and have reduced enthusiasm and motivation for their work (52), which may make them be more inclined to avoid dealing with issues related to mental illness. However, there are no correlation or even negative correlation between age and work experience with mental illness-related stigma in some other studies (24, 53, 54). This paradox may stem from differences in professional roles: specialized mental health professionals might mitigate age-related biases through systematic training, whereas primary healthcare professionals lack such interventions, leading to the accumulation of traditional cognitive stereotypes with prolonged work experience (55). On the other hand, it may be related to the differences in regions, cultures and measurement methods. The protective effect of educational attainment is consistent with previous studies (14, 17), confirming the “knowledge-Attitude Correction Theory” (56), people with lower education levels may be relatively lack of channels and ability to obtain mental health knowledge and mental illness-related information, which is easily affected by social prejudice, which leads to increased mental illness-related stigma. It is worth noting that the influence of marital status may be related to the superimposed pressure of family responsibilities and social expectations in Chinese culture (57). Married PHCPs are more susceptible to stigmatization cognition when fulfilling multiple social roles. Although this study found no statistically significant gender differences, the findings require contextual interpretation through socio-cultural lenses. Evidence suggests that gender’s influence on stigma often interacts with geographical factors: rural female PHCPs may exhibit higher stigmatizing attitudes than their urban counterparts due to reinforced traditional gender roles, while male PHCPs might conceal biases to preserve perceived authority (58, 59). Furthermore, female PHCPs showed higher odds of mental illness-related stigma reduction after contact with mentally ill patients, suggesting gender may indirectly shape attitudes through a “contact-cognitive modification” pathway (60). This mechanism remained unobserved in our study, potentially due to low-quality mental health service contact in rural areas—PHCPs’ interactions are often limited to crisis intervention rather than systematic therapeutic engagement, which may be insufficient to trigger cognitive restructuring (61). This suggests that anti-stigma interventions targeting PHCPs need to adopt a stratified design: 1)for older individuals with low education levels, the focus should be on cognitive restructuring, 2) for married groups, it is necessary to integrate family support systems to reduce role conflicts (62), 3) develop targeted training programs for physicians with over 16.5 years of work experience, emphasizing strategies for the positive transformation of negative experiences, such as burnout management and patient communication skills (63).

In terms of psychology, the results of this study showed that the mental illness-related stigma (scores of MICA) was significantly positively correlated with social distance (SDS), and negatively correlated with mental health knowledge (MAKS), RIBS, contact quantity (CQTS-Work), and contact quality (CQLS). The results of the binary logistic regression analysis showed that social distance had a significant positive impact on mental illness-related stigma among PHCPs, while mental illness-related knowledge and behavioral discrimination had negative impacts on PHCPs’ mental illness-related stigma. However, ROC curve analysis revealed that mental health knowledge had a lower predictive value for mental illness-related stigma, whereas behavioral discrimination and social distance showed higher predictive values. In this study, the cutoff values for MAKS, RIBS, and SDS were 45.5, 11.5, and 13.5, respectively. Values higher or lower than these cutoffs could indicate the presence of mental illness-related stigma among PHCPs. Specifically, PHCPs maintaining greater social distance from patients exhibit heightened mental illness-related stigma levels, which is consistent with previous studies (30, 64). This might stem from the fact that PHCPs may have internalized the stereotype that “mental illness = danger/unpredictability” (39) and limited exposure opportunities in the long-term social and cultural environment, ultimately leading to a reduction in sympathy and understanding for patients (65). On the other hand, insufficient training exacerbates this relationship. When confronted with the complexity of treatment, physicians are more inclined to adopt defensive strategies such as referrals or restrictive prescriptions. This decision-making model further solidifies the vicious cycle of “professional incompetence - distance maintenance - stigma enhancement” (66). Notably, while mental health knowledge demonstrates an inverse correlation with stigmatization (21, 22), this relationship remains contentious: empirical evidence reveals paradoxical patterns where knowledge enhancement may inadvertently reinforce mental illness-related stigma, particularly through stereotyping specific diagnoses (e.g., heightened “dangerousness” perceptions in schizophrenia) (67) or measurement artifacts from instrument limitations (e.g., assessing knowledge breadth while neglecting conceptual depth) (20). Although acquiring stigma-related mental health knowledge theoretically enables PHCPs to better comprehend mental illness etiology and manifestations potentially mitigating prejudice, multiple studies paradoxically report non-significant or even positive correlations between knowledge levels and stigmatization (59, 64). Such inconsistencies may stem from methodological heterogeneity across study populations, assessment tools (e.g., conflating factual recall with critical understanding), and cultural contexts. When discriminatory behaviors are prevalent within a group, individuals may internalize these behaviors as personal attitudes through observational learning (68). This mechanism is evidenced by PHCPs with more severe behavioral discrimination demonstrating higher levels of mental illness-related stigma. In rural China, the behavioral patterns of PHCPs exhibit a unique “protective discrimination” characteristic—manifested through both excessive protective measures like compulsory restraints and the use of derogatory labels such as “lunatics” (30, 69). Notably, this paradoxical phenomenon parallels findings from primary care studies in Chile, where healthcare workers simultaneously engage in discriminatory triage practices while evading responsibilities through institutional avoidance (70). Furthermore, the contact quality and the contact quantity at work are significantly negatively correlated with the mental illness-related stigma, which is consistent with the vast majority of research results (22, 23, 71–73). However, some studies have shown that the level of mental illness-related stigma is not related to the contact quantity (15), and even the long-term exposure of medical staff may exacerbate the stigma due to work stress or negative interaction experiences (30, 51, 52). We further found that the contact quality has a greater correlation on mental illness-related stigma than the contact quantity at work, which substantiates the core hypothesis of the Enhanced Contact Model (ECM) proposed by Ran, which suggests that increasing the level of positive contact should be helpful to reduce mental illness-related stigma (22). However, current research has paid insufficient attention to the heterogeneity of contact effects. For instance, Boyd et al. found that personal contact significantly reduces stigma, while the role of workplace contact remains controversial (65); Aflakseir et al. further noted that only structured workplace contact (e.g., combined with professional training) can reduce stigma, whereas mere frequency accumulation shows no significant effect (74). By distinguishing between quality and quantity dimensions of contact, this study reveals that in primary healthcare settings, the depth of professional interactions (such as empathetic communication and symptom identification training) may better infects changes in mental illness-related stigma levels compared to contact frequency, thereby providing a novel explanatory pathway for contradictory findings. Specifically, reduced workplace contact frequency with persons with mental illness heightens PHCPs’ susceptibility to stereotypes (59), while culturally entrenched negative societal attitudes amplify stigma through dual pathways: 1) Poor-quality interactions hinder clinical understanding of patients’ lived experiences; 2) Limited empathetic engagement perpetuates compassion deficits (75). These findings provide critical targets for developing targeted intervention strategies: it is recommended to prioritize ECM-based training programs for enhancing contact quality concurrently establish a social distance monitoring system, and develop context-specific knowledge assessment tools to mitigate the risk of conceptual oversimplification.

### 4.1 Limitations

This study still has several limitations that need to be noted: 1). This study was a cross-sectional study, unable to determine the causal relationship between variables, and further longitudinal studies should be conducted in the future. 2). The sample size of this study was not very big, and the PHCPs were located in a rural area of Chengdu, China, which may not represent all rural PHCPs in China. Future studies should include larger and more diverse samples from different rural regions to enhance generalizability. 3). The data were collected via self-report questionnaires, which are susceptible to social desirability bias and may be affected by participants’ subjective feelings and biases. These methodological constraints could compromise the accuracy and reliability of the findings. To strengthen validity, future research should consider employing multiple data collection methods—such as behavioral measures, observational studies. 4). MICA demonstrated a Cronbach’s alpha of 0.687, falling below the recommended threshold of 0.7. Future studies should revise the MICA scale with cultural adaptation and validation, and incorporate behavioral experiments or objective indicators to enhance the validity of mental illness-related stigma assessment. 5). This study did not address key potential confounders such as PHCPs’ burnout levels, workplace culture, or prior mental health training, which could influence mental illness-related stigma manifestation, future research needs to take these factors into comprehensive consideration. 6).The study did not explore qualitative aspects of mental illness-related stigma (e.g., narratives from PHCPs) or differentiate between mental illness types (e.g., depression vs. schizophrenia) that may elicit varying stigma levels. Future research should adopt mixed-methods designs combining thematic analysis of PHCPs’ narratives with disorder-specific stigma assessments to capture stigma heterogeneity across mental health conditions.

## 5 Conclusion

This study aimed to explore mental illness-related stigma and its associated factors among PHCPs in a Chinese rural area. The results of the study have indicated the following: 1). The mental illness-related stigma of PHCPs in rural areas of China is serious. 2). PHCPs with longer work experience, greater social distance from persons with mental illness, less mental health knowledge, and stronger behavioral discrimination towards persons with mental illness were more likely to have mental illness-related stigma. These findings will improve the understanding of mental illness-related stigma and its associated factors in PHCPs in rural China. Based on the findings, targeted mental health training and education programs and anti-stigma interventions should be urgently developed for PHCPs for reducing their mental illness-related stigma, improving the quality of community mental health services, and facilitating the treatment and rehabilitation of persons with mental illness in rural area.

## Data Availability

The raw data supporting the conclusions of this article will be made available by the authors, without undue reservation.
